# Elevated systolic pulmonary artery pressure is a substantial predictor of increased mortality after transcatheter aortic valve replacement in males, not in females

**DOI:** 10.1007/s00392-023-02307-z

**Published:** 2023-09-26

**Authors:** Elke Boxhammer, Christiane Dienhart, Joseph Kletzer, Susanne Ramsauer, Kristen Kopp, Erika Prinz, Wilfried Wintersteller, Hermann Blessberger, Matthias Hammerer, Clemens Steinwender, Michael Lichtenauer, Uta C. Hoppe

**Affiliations:** 1 Department of Internal Medicine II, Division of Cardiology, Paracelsus Medical University of Salzburg, Müllner Hauptstraße 48, 5020 Salzburg, Austria; 2 Department of Internal Medicine I, Division of Gastroenterology, Hepathology, Nephrology, Metabolism and Diabetology, Paracelsus Medical University of Salzburg, Salzburg, Austria; 3 Department of Cardiology, Kepler University Hospital, Medical Faculty of the Johannes Kepler University Linz, Linz, Austria

**Keywords:** Aortic valve stenosis, Gender, Pulmonary hypertension, Systolic pulmonary artery pressure, Transcatheter aortic valve replacement

## Abstract

**Background:**

While pulmonary hypertension (PH) in patients with severe aortic valve stenosis (AS) is associated with increased mortality after transcatheter aortic valve replacement (TAVR), there is limited data on gender differences in the effects on long-term survival.

**Objective:**

The aim of this retrospective, multicenter study was to investigate the prognostic impact of pre-interventional PH on survival of TAVR patients with respect to gender.

**Methods:**

303 patients undergoing TAVR underwent echocardiography to detect PH prior to TAVR via measurement of systolic pulmonary artery pressure (sPAP). Different cut-off values were set for the presence of PH. The primary endpoint was all-cause mortality at 1, 3 and 5 years.

**Results:**

Kaplan–Meier analysis by gender showed that only males exhibited significant increased mortality at elevated sPAP values during the entire follow-up period of 5 years (sPAP ≥ 40 mmHg: *p* ≤ 0.001 and sPAP ≥ 50 mmHg: *p* ≤ 0.001 in 1- to 5-year survival), whereas high sPAP values had no effect on survival in females. In Cox regression analysis based on the selected sPAP thresholds, male gender was an independent risk factor for long-term mortality after TAVR in all time courses.

**Conclusion:**

Male gender was an isolated risk factor for premature death after TAVR in patients with echocardiographic evidence of PH and severe AS. This could mean that, the indication for TAVR should be discussed more critically in men with severe AS and an elevated sPAP, while in females, PH should not be an exclusion criterion for TAVR.

**Graphical abstract:**

Graphical abstract of the study (Created with BioRender.com)

Image material of CoreValve™ Evolut™ was kindly provided by © Medtronic Inc.

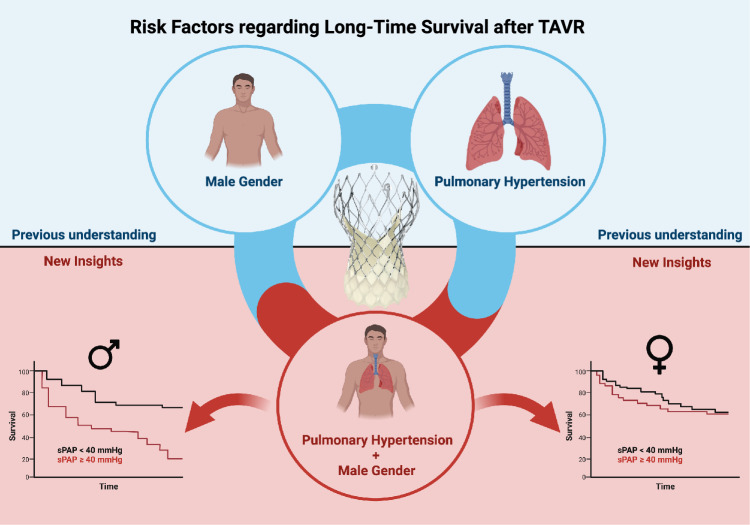

**Supplementary Information:**

The online version contains supplementary material available at 10.1007/s00392-023-02307-z.

## Introduction

Pulmonary hypertension (PH) holds significant importance as a risk determinant in cases of severe aortic valve stenosis (AS) preceding transcatheter aortic valve replacement (TAVR). Its presence adversely influences patient outcomes, linked to elevated risks of fatality and heart failure [1, 2]. Echocardiography stands as the predominant non-invasive technique for detecting PH in severe AS cases. This method offers precise insights into pulmonary artery pressure, right ventricular size and function and left ventricular measurements and operation. The application of Doppler techniques, including the assessment of maximal tricuspid regurgitation velocity (TR Vmax), aids in estimating systolic pulmonary artery pressure (sPAP), serving as a widely utilized clinical approach to approximating PH [3].

TAVR constitutes a minimally invasive intervention for addressing severe AS, garnering extensive acceptance due to its demonstrated enhancements in mortality and morbidity within this cohort [4]. However, the ramifications of PH in patients with severe AS undergoing TAVR remain incompletely grasped. Previous research implies a correlation between PH and escalated mortality and morbidity post-TAVR [5]. Yet, an insufficiency of investigations delves into gender-specific discrepancies in outcomes within this collective. The precise impact of PH on post-TAVR outcomes in male versus female patients with severe AS remains unclear.

Thus, we analysed baseline characteristics, procedural data and clinical outcomes of patients with severe AS and PH who underwent TAVR with respect to gender. This is one of the first studies to examine sex-specific differences in outcomes after TAVR, based on non-invasively determined measures of PH using ultrasound measured sPAP as a proxy. It provides insights into the effect of PH on outcomes after TAVR by gender. The results of this research may be useful in establishing gender-specific guidelines for the management of severe AS and may influence clinical decision making regarding TAVR in this population.

## Material & methods

### Study population

The study population included 303 patients with indication of TAVR at Paracelsus Medical University Hospital, Salzburg and Kepler University Hospital, Linz in the period from 2016 to 2018. Indication for TAVR was made by the interdisciplinary heart team consisting of experienced cardiac surgeons and interventional cardiologists. Inclusion of patients was consecutive and retrospective. Patients with acute cardiac decompensation at the time of transthoracic echocardiography (TTE) or at the time of TAVR, with bicuspid valve or complex congenital heart diseases or, with a history, that might indicate a pre-capillary component of PH (chronic obstructive pulmonary disease (COPD) GOLD 4, idiopathic pulmonary arterial hypertension, chronic thromboembolic PH, interstitial lung disease, or underlying rheumatologic diseases with pulmonary involvement such as scleroderma, lupus erythematosus, etc.) were excluded from the study in advance.

The study protocol was approved by the local ethics committees of Paracelsus Medical University Salzburg (415-E/1969/5–2016) and Johannes Kepler University Linz (E-41–16) and conducted in accordance to principles of the Declaration of Helsinki and Good Clinical Practice. Written informed consent was available from all patients before study inclusion.

### Transthoracic echocardiography

TTE was performed routinely, on average 1–4 weeks before TAVR using either an iE33 or Epiq 5 (Philips Healthcare, Hamburg, Germany) ultrasound device. Examinations were conducted by experienced clinicians with more than 4 years of training in echocardiography. Severe AS was classified according to current guidelines of the European Society for Cardiology (ESC). Left ventricular ejection fraction (LVEF) was calculated using Simpson’s method. To graduate mitral, aortic and tricuspid valve regurgitation in minimal, mild (I), moderate (II) and severe (III) spectral and color-Doppler images were used. TRVmax was obtained by continuous wave Doppler over the tricuspid valve. Pulmonary artery pressure (PAP), right atrial pressure (RAP) and at least sPAP was calculated as described previously [6]. As part of an extensive literature search and a self-authored review on the topic of non-invasive ways of determining PH in severe AS, the most commonly used sPAP cut-off values of 40 and 50 mmHg were used [1, 7, 8]. To also assess the severity of echocardiographically determined PH, patients were further subdivided into no PH by sPAP < 35 mmHg, mild PH by sPAP 35–50 mmHg, moderate PH by sPAP 51–70 mmHg and severe PH by sPAP > 70 mmHg [9]. This classification is based on the recommendations of the American Society for Echocardiography [10, 11].

### TAVR

All 303 patients in this study underwent TAVR using transfemoral access with second (CoreValve™ Evolut™ R; Medtronic Inc., Minneapolis, MN, USA) or third (CoreValve™ Evolut™ Pro; Medtronic Inc., Minneapolis, MN, USA) generation devices. The procedure was performed as previously described [12].

### Statistical analysis

Statistical analysis and graphical representation was performed using SPSS (Version 25.0, SPSSS Inc., USA). Kolmogorov–Smirnov–Lilliefors test was carried out to test variables for normal distribution. Normally distributed metric data were expressed as mean ± standard deviation (SD) and analysed using an unpaired student’s t test. Not-normally distributed metric data were expressed as median and interquartile range (IQR); Mann–Whitney U test was applied for statistical analysis here. Frequencies/percentages were used for categorical data and compared using the chi-squared test. First of all, a Kaplan–Meier curve with corresponding log-rank tests and numbers at risk was generated to determine whether there were differences in 1- to 5-year survival between male and female gender. To also graphically illustrate the impact of echocardiographically measured PH on patient survival, Kaplan–Meier curves with different sPAP values were charted. Univariate Cox proportional hazard regression models in dependence of different sPAP values (≥ 40 mmHg and ≥ 50 mmHg) were used to calculate hazard ratios (HR) and 95% CI for several influencing factors associated with 1-, 3- and 5-year-mortality. For better comparability, a z-transformation was performed for metric data. Afterwards, multivariate Cox regression was performed to assess independent predictors of mortality. Therefore, covariates associated with mortality in the univariate analysis (*p* < 0.100) were entered and a backward variable elimination was performed. Subsequently, the Kaplan–Meier curves were again calculated using the above-mentioned sPAP values and this time strictly separated by gender. A separate univariate and multivariate Cox proportional hazard regression regarding 1-, 3- and 5-year survival as a function of gender was also carried out. Gender-specific area under the receiver operator characteristics (AUROC) curves with area under the curve (AUC) and separate analysis of Youden Index (YI) were performed using different time periods of survival (1, 2, 3, 4 and 5 years) to determine the respective sPAP cut-off value. Ultimately, the categorization of PH severity was determined based on the prevailing guidelines established by the American Society for Echocardiography, classifying it as absent (sPAP < 35 mmHg), mild (sPAP 35–50 mmHg), moderate/severe (sPAP > 50 mmHg). Subsequently, Kaplan–Meier curves were generated for the complete participant group, as well as for the distinct subsets of male and female patients. A *p* value < 0.05 was considered statistically significant.

## Results

### Study cohort and baseline characteristics

303 patients, 151 men and 152 women, were enrolled at Paracelsus Medical University Hospital, Salzburg and Kepler University Hospital, Linz. An overview of the baseline characteristics is provided in Table 1. On average, the subjects were 82.6 ± 4.8 years old. sPAP was ≥ 40 mmHg in 46.9% of patients. Regarding baseline characteristics in males vs. females, our collective was reasonably comparable other than for expected gender differences in height, body weight, hematocrit and hemoglobin. Immediate pre-intervention creatinine was higher in men compared to women (1.1 ± 0.40 mg/dl vs. 0.90 ± 0.40 mg/dl; *p* = 0.003); however, the estimated glomerular filtration rate (eGFR) determined using the CKD-EPI formula and thus corrected for gender and age showed comparable values for renal function. However, men, in contrast to women, had a slightly reduced LVEF (53.5 ± 11.5% vs. 56.6 ± 8.7%; *p* = 0.009) and STS score (2.1 ± 1.3 vs. 3.4 ± 2.2; *p* < 0.001). Men also had an increased rate of post-TAVR pacemaker implantation (29.8% vs. 15.8%; *p* = 0.004) and atrial fibrillation (39.7% vs. 28.9%; *p* = 0.048).

**Table 1:** Baseline characteristics of the study cohort

	Total	Men	Women	*P* value
No. (%)				
Total	303 (100.0)	151 (49.8)	152 (50.2)	0.991
Age				
60–69	6 (2.0)	5 (3.3.)	1 (0.7)	0.097
70–79	70 (23.1)	35 (23.2)	35 (23.0)	0.975
80–89	209 (69.0)	102 (67.5)	107 (70.4)	0.592
≥ 90	18 (5.9)	9 (6.0)	9 (5.9)	0.988
BMI				
< 18.5	3 (1.0)	2 (1.3)	1 (0.6)	0.591
18.5–24.9	135 (44.6)	65 (43.0)	70 (46.1)	0.466
25.0–29.9	108 (35.6)	62 (41.1)	46 (30.3)	0.015
30.0–34.9	42 (13.9)	16 (10.6)	26 (17.1)	0.061
35.0–39.9	14 (4.6)	6 (4.0)	8 (5.3)	0.521
≥ 40.0	1 (0.3)	0 (0.0)	1 (0.6)	0.305
NYHA ≥ III	163 (53.8)	73 (48.3)	90 (58.9)	0.135
Diabetes mellitus	74 (24.4)	33 (21.9)	41 (27.0)	0.300
Arterial hypertension	248 (81.8)	120 (79.5)	128 (84.2)	0.284
CVD	208 (68.6)	111 (73.5)	97 (63.8)	0.097
Previous myocardial infarction	13 (4.3)	4 (2.6)	9 (5.9)	0.156
Atrial fibrillation	104 (34.3)	60 (39.7)	44 (28.9)	0.048
Previous cardiac surgery	17 (5.6)	12 (7.9)	5 (3.3)	0.078
Pacemaker (before TAVR)	18 (5.9)	11 (7.3)	7 (4.6)	0.324
Malignancy	58 (19.1)	35 (23.2)	23 (15.1)	0.075
Stroke (before TAVR)	23 (7.6)	12 (7.9)	11 (7.2)	0.790
PAOD	19 (6.3)	6 (4.0)	13 (8.6)	0.100
COPD	43 (14.2)	25 (16.6)	18 (11.8)	0.240
COPD Gold 1	27 (8.9)	17 (11.3)	10 (6.6)	0.153
COPD Gold 2	11 (3.6)	5 (3.3)	6 (3.9)	0.767
COPD Gold 3	5 (1.7)	3 (2.0)	2 (1.3)	0.647
COPD Gold 4	0 (0.0)	0 (0.0)	0 (0.0)	1.000
LVEF				
≤ 40	33 (10.9)	24 (15.9)	9 (5.9)	0.007
41–49	24 (7.9)	12 (7.9)	12 (7.9)	0.483
≥ 50	246 (81.2)	115 (76.2)	131 (86.2)	0.090
HfpEF	94	39	55	0.238
sPAP				
≥ 40	142 (46.9)	74 (49.0)	68 (44.7)	0.456
≥ 50	75 (24.8)	38 (25.2)	37 (24.3)	0.868
< 35	123 (40.6)	61 (40.4)	62 (40.8)	0.945
35–50	123 (40.6)	56 (37.1)	67 (44.1)	0.215
51–70	45 (14.9)	26 (17.2)	19 (12.5)	0.248
> 70	12 (4.0)	8 (5.3)	4 (2.6)	0.234
AVI ≥ II°	54 (17.9)	22 (14.6)	32 (21.1)	0.164
MVI ≥ II°	78 (25.8)	36 (23.8)	42 (27.6)	0.354
TVI ≥ II°	59 (19.5)	25 (16.6)	34 (22.4)	0.204
Pacemaker (after TAVR)	69 (22.8)	45 (29.8)	24 (15.8)	0.004
Vascular complications	23 (7.6)	12 (7.9)	11 (7.2)	0.815
Stroke (after TAVR)	9 (3.0)	4 (2.6)	5 (3.3)	0.743
Mean ± SD				
Age (years)	82.6 ± 4.8	82.6 ± 5.2	82.6 ± 4.4	0.911
Height (cm)	166.9 ± 8.7	172.9 ± 6.4	160.7 ± 6.0	< 0.001
Weight (kg)	73.8 ± 14.4	78.2 ± 12.8	69.2 ± 14.6	< 0.001
BMI (kg/m^2^)	26.4 ± 4.9	26.1 ± 4.0	26.8 ± 5.8	0.288
eGFR CKD-EPI (ml/min/1.73m^2^)	62.5 ± 17.3	64.8 ± 17.9	60.7 ± 19.4	0.187
LVEF (%)	55.0 ± 10.3	53.5 ± 11.5	56.6 ± 8.7	0.009
LVEDD (mm)	43.6 ± 17.2	45.0 ± 18.6	42.1 ± 15.5	0.270
IVSd (mm)	14.5 ± 3.0	14.8 ± 3.4	14.2 ± 2.5	0.143
AV Vmax (m/s)	4.4 ± 0.9	4.4 ± 1.0	4.3 ± 0.7	0.386
AV dpmax (mmHg)	79.0 ± 19.0	79.5 ± 17.9	78.5 ± 20.1	0.653
AV dpmean (mmHg)	48.1 ± 12.0	48.0 ± 11.3	48.2 ± 12.6	0.889
TAPSE (mm)	22.1 ± 4.4	22.4 ± 4.5	21.7 ± 4.3	0.320
sPAP (mmHg)	37.1 ± 19.1	38.8 ± 19.1	35.5 ± 18.9	0.123
Median ± IQR				
STS score	2.8 ± 1.9	2.1 ± 1.3	3.4 ± 2.2	< 0.001
Creatinine (mg/dl)	1.0 ± 0.4	1.1 ± 0.40	0.9 ± 0.4	0.003
BNP (pg/ml)	1443.0 ± 2460.5	1918.0 ± 3460.7	1240.0 ± 1643.4	0.127
HK (%)	38.0 ± 6.4	39.1 ± 6.9	37.4 ± 6.0	0.002
HB (g/dl)	12.7 ± 2.3	13.4 ± 2.4	12.4 ± 1.9	< 0.001
CK (U/l)	76.0 ± 61.5	79.0 ± 69.0	73.5 ± 51.8	0.069

*BMI* body mass index; *CVD* cardiovascular disease; *PAOD* peripheral arterial occlusive disease; *COPD* chronic obstructive pulmonary disease; *LVEF* left ventricular ejection fraction; *HFpEF* heart failure with preserved ejection fraction; *sPAP* systolic pulmonary artery pressure; *TAVR* transcatheter aortic valve replacement; *LVEDD* left ventricular end-diastolic diameter at diastole; *IVSd* interventricular septal thickness at diastole; *AV Vmax*: maximal velocity over aortic valve; *AV dpmax* maximal pressure gradient over; *AV dpmean* mean pressure gradient over aortic valve; *TAPSE* tricuspid annular plane systolic excursion; *sPAP* systolic pulmonary artery pressure; *AVI* aortic valve insufficiency; *MVI* mitral valve insufficiency; *TVI* tricuspid valve insufficiency; *eGFR* estimated glomerular filtration rate; *BNP* brain natriuretic peptide; *HK* hematocrit; *HB* hemoglobin; *CK* creatine kinase

### Short- and long-term survival regarding gender

**Fig. 1:**
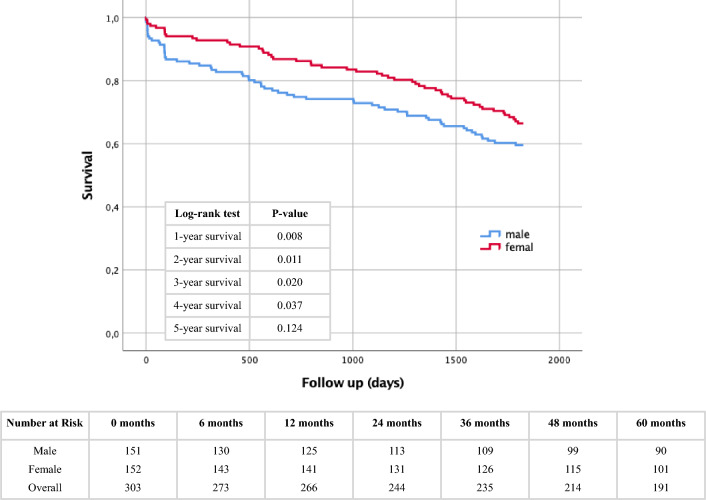
Kaplan–Meier curve with corresponding numbers at risk and annually log-rank tests for detection of 1- to 5-year survival in dependence of gender

To identify gender-specific differences in long-term survival after TAVR, Kaplan–Meier curves were generated up to 5 years after TAVR with corresponding log-rank tests and numbers at risk calculated annually (Fig. 1). There was a significantly higher mortality in males in the first 4 years after intervention. Only in the 5th year after TAVR, the mortality men and women were similar in mortality (log-rank test 5-year survival: *p* = 0.124).

### Short- and long-term survival regarding sPAP

**Fig. 2:**
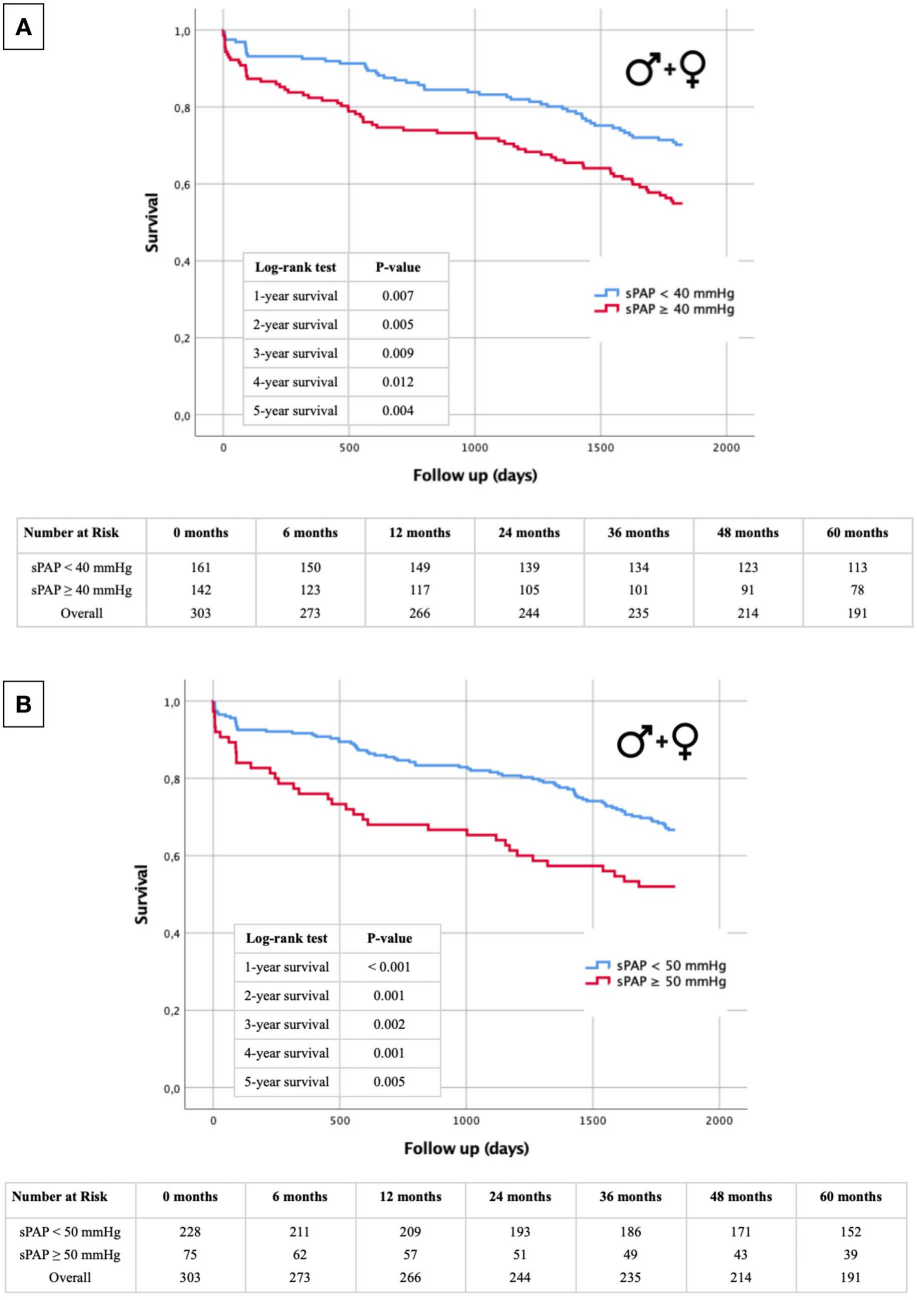
Kaplan–Meier curve with corresponding numbers at risk and annually log-rank tests for detection of 1- to 5-year survival. **A**: sPAP < 40 mmHg vs. an sPAP ≥ 40 mmHg. **B**: sPAP < 50 mmHg vs. an sPAP ≥ 50 mmHg

To assess the sex-independent effect of PH on survival in the present collective, Kaplan–Meier curves were generated with different sPAP cut-off values (≥ 40 mmHg and ≥ 50 mmHg). Overall, an elevated sPAP, regardless of the cut-off selected, significantly increased mortality rates throughout the 5-year recording period in our cohort, although we would argue this is driven by the elevated mortality in males. Detailed statistical data analysis with log-rank tests and numbers at risk are shown in the corresponding figures (Fig. 2A: sPAP ≥ 40 mmHg; Fig. 2B: sPAP ≥ 50 mmHg).

### sPAP-dependent mortality risk factors after TAVR

To investigate influencing factors concerning mortality after TAVR in dependence of different sPAP values (≥ 40 mmHg and ≥ 50 mmHg), a univariate and multivariate Cox proportional hazard regression was performed (Table 2: 1-year mortality; Table 3: 3-year mortality; Table 4: 5-year mortality). Male sex was consistently a significant, and in some cases highly significant, factor for premature death in the univariate analysis, regardless of the observation period and regardless of the sPAP cut-off value. After inclusion of further clinical characteristics with a *p* < 0.100 in a multivariate analysis, male sex remained an independent risk factor for estimation of mortality after 1, 3 and 5 years in all calculations.

**Table 2:** Univariate and multivariate cox regression analysis detecting 1-year mortality in dependence of different sPAP values (only characteristics included showing a *p* ≤ 0.100 in univariate analysis)

Cox regression analysis	Univariate		Multivariate	
	Hazard ratio (95% CI)	*p* value	Hazard ratio (95% CI)	*p* value
1-year mortality sPAP ≥ 40 mmHg				
Gender (male)	5.477 (1.879–15.965)	0.002	9.546 (2.218–41.096)	0.002
Height	1.676 (1.091–2.577)	0.019	1.026 (0.458–2.295)	0.951
Weight	1.589 (1.078–2.342)	0.019	1.082 (0.659–1.776)	0.755
Previous cardiac surgery	5.823 (2.179–15.560)	< 0.001	3.863 (1.291–11.555)	0.016
Stroke (before TAVR)	2.464 (0.845–7.183)	0.099	2.048 (0.575–7.296)	0.269
LVEF	0.695 (0.514–0.940)	0.018	0.858 (0.548–1.344)	0.504
AV dpmax	0.704 (0.479–1.034)	0.074	0.885 (0.331–2.364)	0.808
AV dpmean	0.672 (0.436–1.036)	0.072	0.707 (0.439–1.137)	0.152
Pacemaker (after TAVR)	1.996 (0.882–4.518)	0.097	1.942 (0.798–4.727)	0.144
1-year mortality sPAP ≥ 50 mmHg				
Gender (male)	5.885 (1.701–20.356)	0.005	77.954 (5.268–1153.475)	0.002
Height	1.697 (1.043–2.763)	0.033	0.973 (0.234–4.048)	0.970
Weight	1.704 (1.014–2.862)	0.044	1.183 (0.536–2.611)	0.677
Previous myocardial infarction	5.259 (1.195–23.142)	0.028	34.232 (4.152–282.267)	0.001
Atrial fibrillation	0.311 (0.102–0.946)	0.040	0.282 (0.067–1.191)	0.085
Previous cardiac surgery	5.567 (1.970–15.730)	0.001	0.721 (0.127–4.089)	0.712
AV Vmax	0.331 (0.148–0.737)	0.007	1.557 (0.011–230.508)	0.862
AV dpmax	0.700 (0.480–1.022)	0.065	0.394 (0.215–0.721)	0.003
AV dpmean	0.645 (0.407–1.020)	0.061	0.850 (0.292–2.476)	0.766
TVI ≥ II°	0.282 (0.082–0.973)	0.045	1.323 (0.196–8.928)	0.774
Pacemaker (after TAVR)	2.986 (1.154–7.728)	0.024	8.723 (2.135–35.644)	0.003

*sPAP* systolic pulmonary artery pressure; *TAVR* transcatheter aortic valve replacement; *LVEF* left ventricular ejection fraction; *AV dpmax* maximal pressure gradient over aortic valve; *AV dpmean* mean pressure gradient over aortic valve; *AV Vmax* maximal velocity over aortic valve; *TVI* tricuspid valve insufficiency

**Table 3:** Univariate and multivariate cox regression analysis detecting 3-year mortality in dependence of different sPAP values (only characteristics included showing a *p* ≤ 0.100 in univariate analysis)

Cox regression analysis	Univariate		Multivariate	
	Hazard ratio (95% CI)	*p* value	Hazard ratio (95% CI)	*p* value
3-year mortality sPAP ≥ 40 mmHg				
Gender (male)	4.007 (1.911–8.401)	< 0.001	4.210 (1.843–9.618)	0.001
Height	1.525 (1.095–2.124)	0.012	0.771 (0.470–1.264)	0.302
Weight	1.333 (0.972–1.829)	0.074	1.001 (0.660–1.517)	0.998
Previous cardiac surgery	7.137 (3.095–16.455)	< 0.001	5.231 (2.143–12.769)	< 0.001
LVEF	0.743 (0.580–0.951)	0.019	0.976 (0.720–1.324)	0.877
AV dpmean	0.759 (0.550–1.048)	0.094	0.828 (0.600–1.143)	0.251
3-year mortality sPAP ≥ 50 mmHg				
Gender (male)	4.303 (1.724–10.740)	0.002	17.035 (3.857–75.246)	< 0.001
Height	1.432 (0.958–2.139)	0.080	0.687 (0.377–1.251)	0.219
Previous myocardial infarction	3.722 (0.874–15.860)	0.076	21.218 (3.294–136.692)	0.001
Atrial fibrillation	0.464 (0.202–1.069)	0.071	0.727 (0.395–1.338)	0.306
Previous cardiac surgery	5.768 (2.263–14.702)	< 0.001	2.958 (0.801–10.917)	0.104
AV Vmax	0.401 (0.201–0.799)	0.009	0.313 (0.144–0.678)	0.003
TVI ≥ II°	0.401 (0.161–1.000)	0.050	0.800 (0.269–2.379)	0.688
Pacemaker (after TAVR)	2.293 (0.993–5.294)	0.052	5.517 (1.873–16.254)	0.002

*sPAP* systolic pulmonary artery pressure; *LVEF* left ventricular ejection fraction; *AV dpmean* mean pressure gradient over aortic valve; *AV Vmax* maximal velocity over aortic valve; *TVI* tricuspid valve insufficiency, *TAVR* transcatheter aortic valve replacement;

**Table 4:** Univariate and multivariate cox regression analysis detecting 5-year mortality in dependence of different sPAP values (only characteristics included showing a *p* ≤ 0.100 in univariate analysis)

Cox regression analysis	Univariate		Multivariate	
	Hazard ratio (95% CI)	*p* value	Hazard ratio (95% CI)	*p* value
5-year mortality sPAP ≥ 40 mmHg				
Gender (male)	2.350 (1.401–3.942)	0.001	2.555 (1.416–4.613)	0.002
Height	1.257 (0.970–1.629)	0.084	0.726 (0.486–1.085)	0.119
Previous cardiac surgery	5.482 (2.452–12.257)	< 0.001	8.431 (3.200–22.212)	< 0.001
LVEF	0.800 (0.651–0.982)	0.033	0.841 (0.657–1.077)	0.170
AV dpmax	0.785 (0.605–1.020)	0.070	0.755 (0.537–1.061)	0.106
Stroke (after TAVR)	3.579 (0.865–14.811)	0.078	2.397 (0.402–14.306)	0.338
5-year mortality sPAP ≥ 50 mmHg				
Gender (male)	2.455 (1.240–4.861)	0.010	7.609 (2.857–20.268)	< 0.001
Previous myocardial infarction	5.623 (1.680–18.815)	0.005	16.235 (3.596–73.300)	< 0.001
Atrial fibrillation	0.441 (0.217–0.896)	0.024	0.384 (0.156–0.942)	0.037
Previous cardiac surgery	4.579 (1.864–11.247)	0.001	2.128 (0.570–7.950)	0.262
AV Vmax	0.452 (0.241–0.846)	0.013	0.962 (0.293–3.162)	0.949
AV dpmax	0.736 (0.552–0.981)	0.037	0.524 (0.377–0.729)	< 0.001
Pacemaker (after TAVR)	2.071 (0.996–4.307)	0.051	4.413 (1.766–11.030)	0.001

*sPAP* systolic pulmonary artery pressure; *LVEF* left ventricular ejection fraction; *AV dpmax* maximal pressure gradient over aortic valve; *TAVR* transcatheter aortic valve replacement; *AV Vmax* maximal velocity over aortic valve

### Short- and long-term survival regarding sPAP and gender

**Fig. 3:**
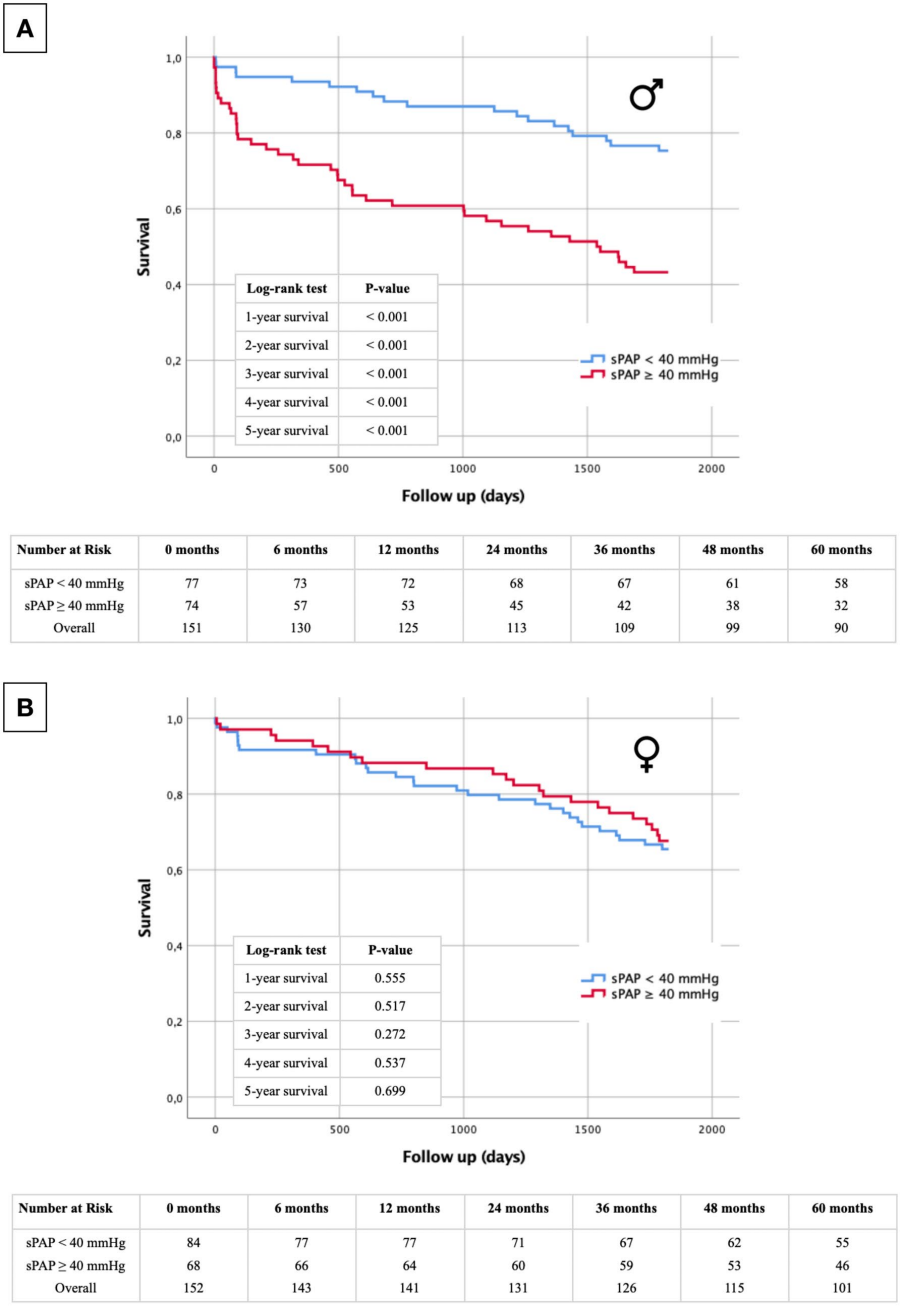
Kaplan–Meier curve with corresponding numbers at risk and annually log-rank tests for detection of 1- to 5-year survival in dependence of an sPAP < 40 mmHg vs. an sPAP ≥ 40 mmHg and in dependence of gender (**A**: male; **B**: female)

**Fig. 4:**
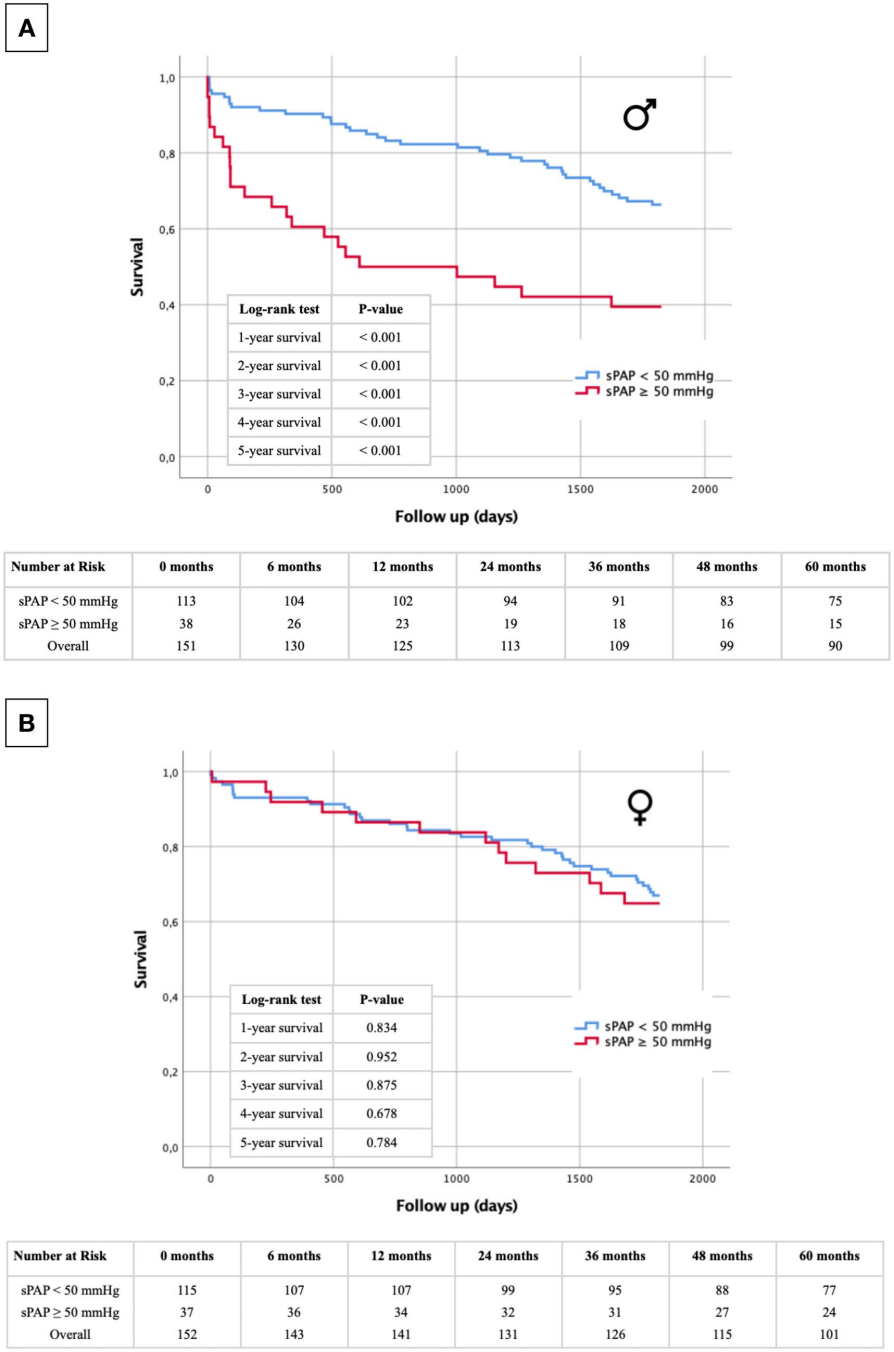
Kaplan–Meier curve with corresponding numbers at risk and annually log-rank tests for detection of 1- to 5-year survival in dependence of an sPAP < 50 mmHg vs. an sPAP ≥ 50 mmHg and in dependence of gender (**A**: male; **B** female)

Kaplan–Meier curves with the selected cut-off values were finally further separated according to male and female gender (Fig. 3A: sPAP ≥ 40 mmHg male, Fig. 3B: sPAP ≥ 40 mmHg female; Fig. 4A: sPAP ≥ 50 mmHg male, Fig. 4B: sPAP ≥ 50 mmHg female). At all of the analysed sPAP levels (≥ 40 mmHg and ≥ 50 mmHg), the log-rank test was highly significant at every time point (1–5 year survival) for premature death, for males only. In contrast, there was no statistically significant differences in long-term survival in female study participants, regardless of sPAP cut-off values based on echocardiography at any timepoint.

### Gender-dependent mortality risk factors after TAVR

In addition to different sPAP values, univariate and multivariate Cox hazard regression analysis depending on gender were also analysed (Table 5). In males, the sPAP value was an independent, highly significant risk factor for 1- and 3-year mortality after TAVR (*p* ≤ 0.001). After 5 years, sPAP still showed a similar trend, although not statistically significant (*p* = 0.093). In females, creatine kinase (CK) was an independent factor for earlier death after TAVR in all analysed periods (1, 3 and 5 years).

**Table 5:** Univariate and multivariate cox regression analysis detecting 1-, 3- and 5-year mortality in dependence of gender (only characteristics included showing a *p* ≤ 0.100 in univariate analysis)

Cox regression analysis	Univariate		Multivariate	
	Hazard ratio (95% CI)	*p* value	Hazard ratio (95% CI)	*p* value
1-year mortality male				
sPAP	2.041 (1.410–2.953)	< 0.001	2.310 (1.568–3.402)	< 0.001
Stroke (after TAVR)	3.522 (0.832–14.919)	0.087	12.609 (2.496–63.708)	0.002
1-year mortality female				
HK	0.564 (0.312–1.019)	0.058	0.621 (0.317–1.217)	0.165
HB	0.565 (0.309–1.034)	0.064	1.643 (0.162–16.628)	0.674
CK	1.243 (1.087–1.420)	0.001	1.265 (1.103–1.450)	0.001
Pacemaker (after TAVR)	3.350 (0.980–11.449)	0.054	3.134 (0.779 –12.611)	0.108
3-year mortality male				
sPAP	1.871 (1.392–2.516)	< 0.001	1.774 (1.293–2.432)	< 0.001
BNP	1.227 (1.001–1.504)	0.049	0.982 (0.754–1.278)	0.890
HB	0.782 (0.583–1.048)	0.099	0.991 (0.680–1.444)	0.962
3-year mortality female				
STS score	1.654 (0.922–2.966)	0.091	1.507 (0.716–3.169)	0.280
LVEF	0.694 (0.461–1.045)	0.080	0.622 (0.229–1.690)	0.352
CK	1.243 (1.089–1.420)	0.001	12.169 (1.020–145.170)	0.048
5-year mortality male				
Age	1.257 (0.980–1.612)	0.072	1.061 (0.764–1.473)	0.730
STS score	1.473 (0.991–2.190)	0.055	0.994 (0.456–2.167)	0.988
sPAP	1.612 (1.258–2.065)	< 0.001	1.405 (0.945–2.088)	0.093
BNP	1.233 (1.027–1.482)	0.025	0.854 (0.558–1.308)	0.469
HK	0.782 (0.609–1.004)	0.054	0.877 (0.258–2.976)	0.833
HB	0.791 (0.619–1.010)	0.060	0.818 (0.515–1.301)	0.396
5-year mortality female				
Previous cardiac surgery	3.116 (0.968–10.026)	0.057	2.911 (0.866–9.789)	0.084
IVSd	1.334 (0.967–1.840)	0.079	1.351 (0.961–1.899)	0.083
CK	1.240 (1.084–1.418)	0.002	1.269 (1.101–1.463)	0.001
Stroke (after TAVR)	4.805 (1.891–12.209)	0.001	6.751 (2.327–19.591)	< 0.001

*sPAP* systolic pulmonary artery pressure; *TAVR* transcatheter aortic valve replacement; *HK* hematocrit; *HB* hemoglobin; *CK* creatine kinase; BNP: brain natriuretic peptide; *LVEF* left ventricular ejection fraction; *IVSd* interventricular septal thickness at diastole

### Gender-specific AUROC analysis of sPAP values for predicting survival after TAVR

**Fig. 5:**
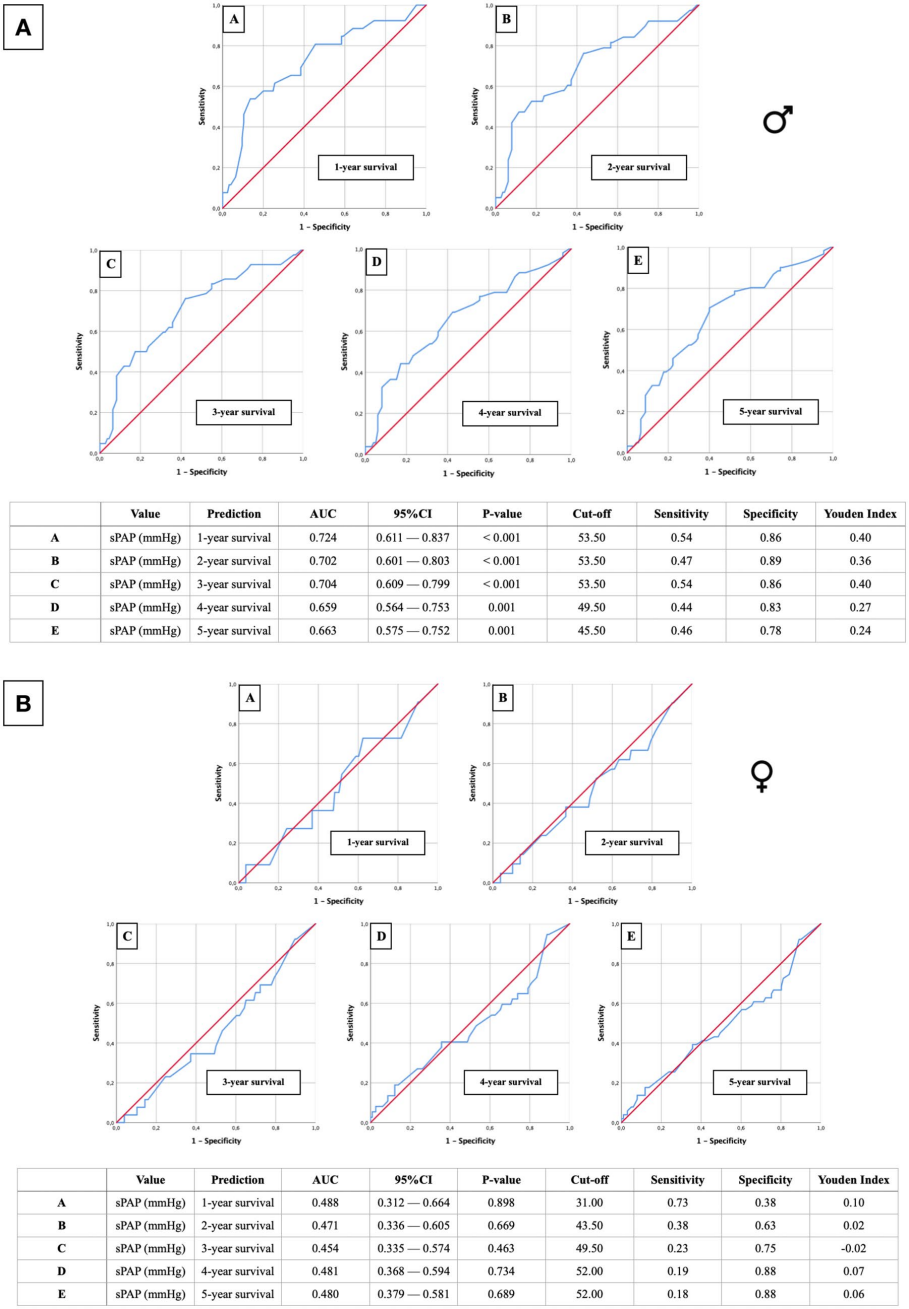
AUROC analyses of sPAP for prediction of 1- to 5-year survival with concerning cut-off values, Youden Index, sensitivity and specificity in dependence of gender (Fig. 5A: male; Fig. 5B: female)

Gender-dependent AUROC analyses (Fig. 5) were calculated to assess sPAP cut-off values in relation to 1- to 5-year survival. In males (Fig. 5A), an sPAP value ≥ 53.50 mmHg was a highly significant, relevant predictor (*p* < 0.001) of premature death after interventional valve replacement in the first 3 years after TAVR. Later, at 4 and 5 years, sPAP values of ≥ 49.50 mmHg (4 years) and ≥ 45.50 mmHg (5 years) were also still highly significant (*p* = 0.001). In contrast, in female gender (Fig. 5B), the AUROC results showed no relevant significance.

### Short- and long-term survival regarding severity of PH—subgroup analysis

**Fig. 6:**
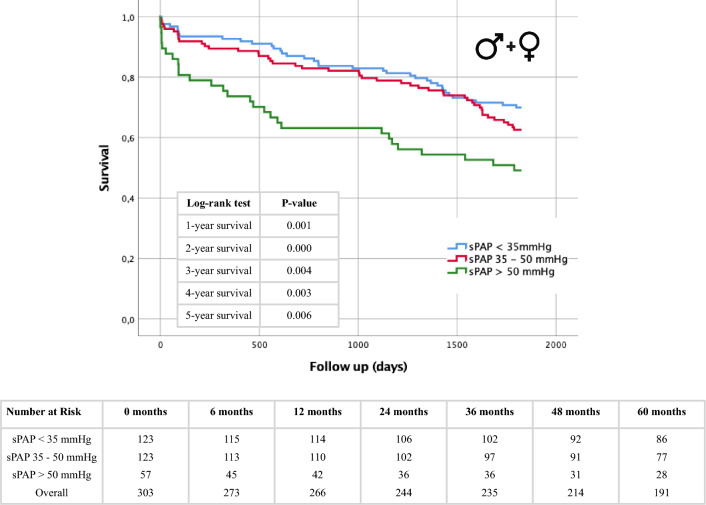
Kaplan–Meier curve with corresponding numbers at risk and annually log-rank tests for detection of 1- to 5-year survival in dependence of PH being absent (sPAP < 35 mmHg), mild (sPAP 35–50 mmHg) or moderate/severe (sPAP > 50 mmHg)

**Fig. 7:**
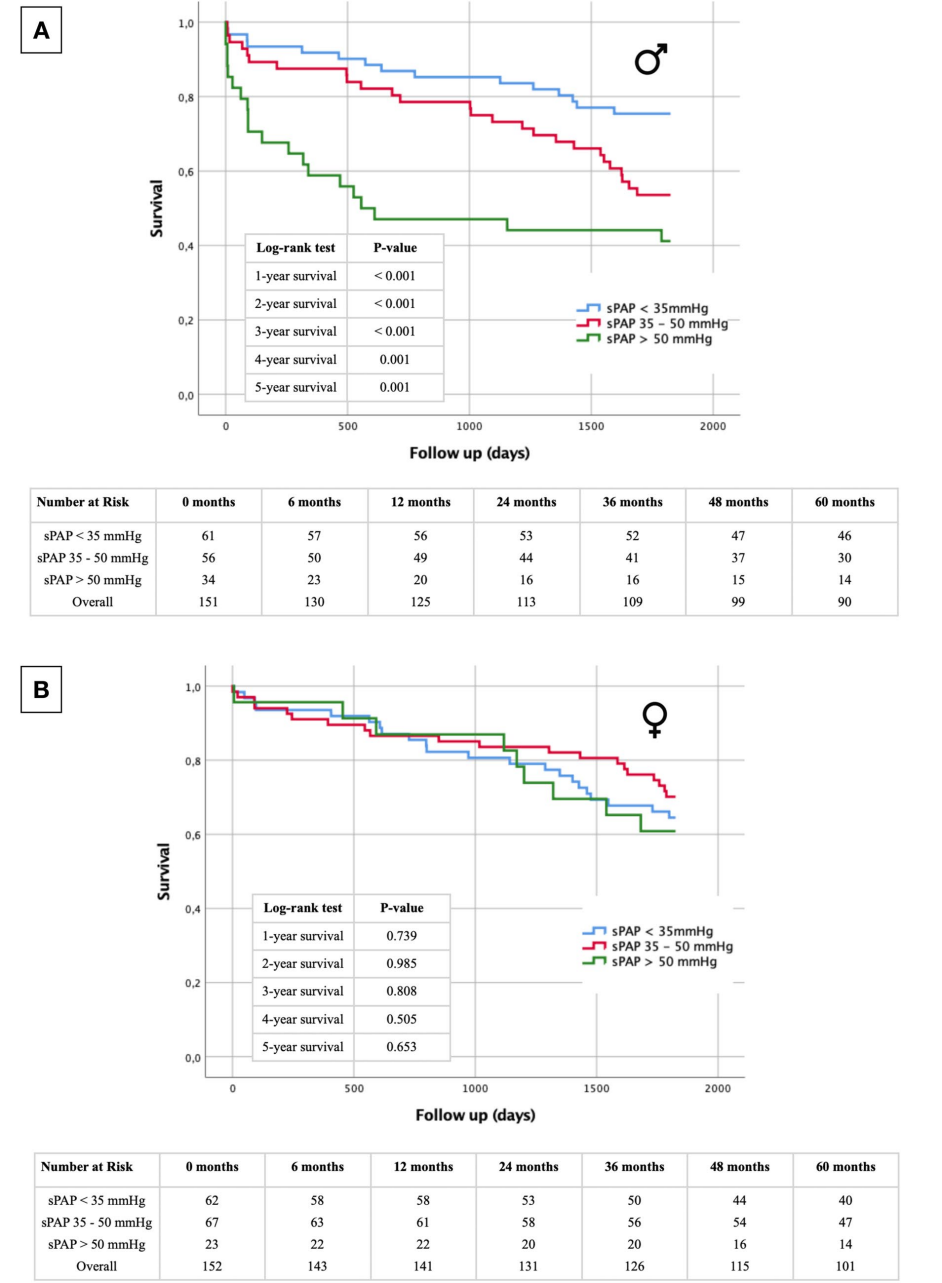
Kaplan–Meier curve with corresponding numbers at risk and annually log-rank tests for detection of 1- to 5-year survival in dependence of PH being absent (sPAP < 35 mmHg), mild (sPAP 35–50 mmHg) or moderate/severe (sPAP > 50 mmHg) and in dependence of gender (**A**: male; **B**: female)

Ultimately, to incorporate the presently accepted echocardiographic categorization of PH, aligning with the recommendations of the American Society of Echocardiography, Kaplan–Meier curves were computed. These calculations encompassed the aforementioned subgroups within the entire cohort (Fig. 6) and were further stratified based on gender (Fig. 7). It is worth noting that due to the limited count of patients exhibiting sPAP values exceeding 70 mmHg (Table 1), the categories of moderate and severe PH were merged into a singular group. In the complete study population (Fig. 6), the most notable risk of premature mortality subsequent to TAVR was evident among patients exhibiting an sPAP exceeding 50 mmHg. The statistical evaluation using log-rank tests revealed significant disparities across all observed survival intervals. Further examination within the male subgroup (Fig. 7A) consistently demonstrated noteworthy distinctions among the different sPAP categories, wherein the cohort with the highest sPAP levels also displayed the highest fatality rates. Conversely, in the female cohort (Fig. 7B), discernible differences in mortality among the varying sPAP classifications were not observed.

## Discussion

This work is the first gender-specific study of TAVR patients to address long-term mortality in the setting of concomitant, non-invasively detected PH using sPAP as a proxy. Our results show that while in males, concomitant elevated sPAP, regardless of cut-off value chosen, is an isolated risk factor for premature death after TAVR in the presence of severe AS, this association was not observed in females study participants.

### Male gender is a risk factor for long-term survival after TAVR

Gender-focused investigations exploring disparities in survival outcomes following TAVR have been plentiful in recent literature. Particularly concerning 1-year survival post-TAVR, our study aligns with findings indicating elevated male mortality at this juncture. Zahn et al. (2013) [13] exhibited a markedly increased 1-year mortality in males (23.6%) compared to females (17.3%) in a cohort of 1391 patients from the German Transcatheter Aortic Valve Interventions Registry (*p* < 0.001). Denegri et al. [14], examining a 3,821-patient Italian multicenter cohort, reported a 1-year mortality of 15.0% in males versus 11.5% in females (*p* < 0.001). Similarly, Yousif et al. [15] observed significantly higher 1-year mortality in males versus females (18.7% vs. 11.7%, *p* = 0.037) within the Swiss TAVI Cohort collective (*n* = 546). This gender-based disparity in survival was not confined to just 1 year; it extended to 3 and 5 years post-TAVR. Denegri et al. [14] undertook a 3-year mortality analysis in the aforementioned Italian cohort, uncovering earlier mortality in males (19.8% vs. 24.9%; *p* < 0.001) after adjusting for baseline characteristics. In 2017, Zahn et al. [16] demonstrated that female gender served as a protective, non-modifiable factor (HR 0.66, 95% CI 0.56–0.77; *p* < 0.001) for 5-year survival after TAVR based on the aforementioned registry's 5-year follow-up of 1444 patients. Our study's determination of significantly elevated 1-year mortality rates (17.2% for males vs. 7.2% for females; *p* = 0.008) seamlessly aligns with previous findings. Our results also revealed statistically significant disparities favoring female gender in 2-year (25.2% vs. 13.8%; *p* = 0.011), 3-year (27.8% vs. 17.1%; *p* = 0.020), and 4-year mortalities (34.4% vs. 23.0%; *p* = 0.037). Many studies have grappled with explaining the heightened male mortality post-TAVR. A common assertion attributes worse baseline vascular comorbidities to male sex across these studies [17]. However, this did not hold true in our analysis. Furthermore, the broader reduced life expectancy in males alone cannot entirely account for the recurrently documented early male mortality post-TAVR. Instead, our study bolsters the hypothesis proposed by Yousif et al. [15], focusing on the idea that the remodeling processes occurring in severe AS due to chronic pressure loading exhibit gender-specific differences. Female patients appear to tolerate left ventricular concentric hypertrophy secondary to AS for a longer duration, which contrasts with males and their propensity for eccentric, partially dilated cardiomyopathy. This notion is backed by the notably lower LVEF before TAVR in males, with nearly identical intraventricular septal thickness and a larger left ventricular end-diastolic diameter. A higher prevalence of atrial fibrillation in males further supports this idea. Hence, it is plausible that the cardiac remodeling process, transitioning from concentric pressure load to eccentric volume load in severe AS, occurs more swiftly and frequently in males, culminating in escalated risk of cardiac contractility loss, consequential heart failure and untimely demise [18;19]. Lastly, an argument could be posited that men present with a more advanced disease state at the time of TAVR, as evidenced by the lower LVEF prior to the procedure, potentially depleting cardiac adaptive capacity in males.

### PH is a risk factor for long-term survival after TAVR

Concomitant post-capillary PH in severe AS is a notable risk factor for heightened post-TAVR mortality, a consensus evident across multiple studies involving invasive right heart catheterization. Schewel et al. [20] observed that patients (*n* = 1400) devoid of PH exhibited notably lower 1-year and 4-year mortality rates compared to those with PH (1-year mortality: 13.8% vs. 22.4%, *p* < 0.001; 4-year mortality: 37.2% vs. 51.5%, *p* < 0.001). In recent times, however, the routine use of invasive right heart catheterization, the gold standard for PH diagnosis, prior to TAVR has diminished. For many TAVR-equipped cardiology centers, a non-invasive assessment using TTE to establish PH presence or absence before intervention suffices. In this regard, sPAP predominantly serves in clinical practice, factoring in the patient's current volume status. Various sPAP cut-off values have been applied in comparative studies to define PH [21, 22]. This study, too, adopts the commonly used thresholds of 40 and 50 mmHg. Past analyses employing the FRANCE-2 registry [23] indicated that patients with severe AS and sPAP ≥ 40 mmHg or ≥ 60 mmHg (No PH: 22%; PH ≥ 40 mmHg and PH ≥ 60 mmHg: 28% each; *p* = 0.032) experienced significantly curtailed 1-year survival after TAVR. Similarly, Barbash et al. [24] documented a similar trend with sPAP ≥ 50 mmHg, highlighting that patients with moderate/severe PH (sPAP ≥ 50 mmHg) had significantly earlier 1-year mortality (*p* = 0.020) compared to those with no/mild PH (sPAP < 50 mmHg). Drawing from their multicenter registry, D’Ascenzo et al. [25] identified sPAP ≥ 40 mmHg as an independent risk factor for all-cause mortality. Bishu et al. [26] demonstrated that sPAP ≥ 50 mmHg was an independent predictor of long-term mortality post-TAVR. Our current study further underscores elevated mortality rates in severe AS patients with verified PH through TTE, irrespective of the chosen pre-TAVR sPAP cut-off value. Thus, sPAP, a clinically straightforward measurement, emerges not just as a valuable tool for non-invasive PH assessment, but also as a clinical parameter apt for pre-TAVR risk stratification.

### PH is a risk factor for long-time survival after TAVR, but only in males

Our study is the first to highlight gender-specific differences in survival risk after TAVR based on echocardiographic evidence of PH prior to intervention. For patients with PH, male gender was shown to be an isolated risk factor for premature death after TAVR. In contrast, in females, concomitant PH had no influence on postinterventional mortality regardless of sPAP cut-off values. While average sPAP was slightly higher in males (38.8 ± 19.1 mmHg) in contrast to females (35.5 ± 18.9 mmHg) in our cohort, this was without statistical significance (*p* = 0.123). Similarly, severity of PH using sPAP as a proxy, was not statistically significantly different (*p* = 0.109) although sPAP values ≥ 60 mmHg were more prevalent in percentage terms in males in our cohort (15.2% vs. 9.2%). Further breakdown of baseline characteristics according to corresponding sPAP groupings (data available on request) did not yield significant gender-specific differences for an sPAP ≥ 40 mmHg for atrial fibrillation (male: 47.5% vs. female: 36.8%, *p* = 0.204) or for mitral regurgitation ≥ II° (male: 26.7% vs. female 38.8%, *p* = 0.120) as a potential additive cause for severe PH. Thus, the question still remains: why is PH, as determined by sPAP, associated with such significantly increased mortality after TAVR, but, only in males? Here, a review by Rodiguez-Arias et al. [27] provides a potential theory, as the cause is also likely to be found in the more rapid remodeling and poorer adaptation of the right ventricle (RV) to pressure or volume loading in males. This hypothesis is supported primarily by the work of Melenovsky et al. [28] and Ventetuolo et al. [29]. Melenovsky and colleagues [28] demonstrated a relevant association between male sex, PH and RV dysfunction using a heart failure with preserved ejection fraction (HFpEF) collective. Ventetuolo et al. [29], using 15,464 veterans with invasive right heart catheterization data, showed that women, in contrast to men, had better survival rates in the presence of PH, regardless of cause, despite higher pulmonary vascular resistance and increased pulmonary artery pulse pressure values. As a potential explanation, the authors suggested a better RV adaptation in females. The question regarding PH reversibility after interventional valve replacement is still open, because in contrast to comparative studies [30, 31], sPAP changes after TAVR were not included in this study. In both Alushi et al. and Masri et al. TAVR patients were divided into different groups according to postinterventional course of sPAP. 51% [30] and 55% [31] of male subjects showed improvement in PH and thus potential reversibility. Whether and to what extent improved sPAP after TAVR could also positively influence male survival needs to be shown in follow-up studies.

## Conclusion

Male gender was shown to be an isolated risk factor for premature death after TAVR in patients with echocardiographic evidence of PH and severe AS. In a clinical setting, this could mean that, the indication for TAVR should be discussed more critically in men with PH and especially an sPAP ≥ 50 mmHg while in females, PH, as defined by an elevated sPAP should not be a definitive exclusion criterion for TAVR according to these study results.

## Limitation

The present, retrospective study design is based on data from a small cohort (*n* = 303; 151 male – 152 female) over a circumscribed time period (2016–2018). A calculation performed to determine the sample size (use of G*Power 3.1—test family: *t* test; statistical test: means; type of power analysis: a priori) provided an optimal sample size of 210 patients per gender for this study using an effect size *d* of 0.5, an alpha error of 0.05, a power (1 minus beta error) of 0.95 and an allocation ratio of 1. The present sample size of approximately 150 patients per gender corresponds to a satisfactory power of 0.86 using the above parameters. Technical pitfalls in echocardiography which might lead to misclassifications cannot be completely excluded, even if examinations were performed by experienced clinical investigators. Furthermore, invasive right heart catheterization, the gold standard for accurate diagnosis regarding the origin of PH (pre-capillary vs. post-capillary) was neither performed in Salzburg nor in Linz, because it is no longer a routine, diagnostic procedure before TAVR. Thus, despite exclusion of obvious factors for pre-capillary PH, our cohort is not guaranteed to consist of only left heart-related, post-capillary PH patients.

### Supplementary Information

Below is the link to the electronic supplementary material.

Supplementary file1 (PDF 115 KB)

Supplementary file2 (PDF 115 KB)

Supplementary file3 (PDF 116 KB)

Supplementary file4 (PDF 116 KB)

Supplementary file5 (PDF 116 KB)

Supplementary file6 (PDF 116 KB)

## Data Availability

Raw data are available from the authors upon request.

## Abbreviations

AS: Aortic valve stenosis
AUC: Area under the curve
AUROC: Area under the receiver operator characteristics
AV Vmax: Maximal velocity over aortic valve
AV dpmax: Maximal pressure gradient over aortic valve
AV dpmean: Mean pressure gradient over aortic valve
CI: Confidence interval
COPD: Chronic obstructive pulmonary disease
eGFR: Estimated glomerular filtration rate
ESC: European Society for Cardiology
HFpEF: Heart failure with preserved ejection fraction
HR: Hazard ratio
IQR: Interquartile range
LVEF: Left ventricular ejection fraction
PAP: Pulmonary artery pressure
PH: Pulmonary hypertension
RAP: Right atrial pressure
RV: Right ventricle
SD: Standard deviation
sPAP: Systolic pulmonary artery pressure
TAVR: Transcatheter aortic valve stenosis
TR Vmax: Maximal tricuspid regurgitation velocity
TTE: Transthoracic echocardiography
YI: Youden index
