# Supplementary material for: Elevated systolic pulmonary artery pressure is a substantial predictor of increased mortality after transcatheter aortic valve replacement in males, not in females
Source: Clin Res Cardiol. 2023 Sep 26;113(1):138–55. doi: 10.1007/s00392-023-02307-z (PMC10808322; doi:10.1007/s00392-023-02307-z)
Supplement: Supplementary file 1 — Supplementary file1 (PDF 115 KB) [file 392_2023_2307_MOESM1_ESM.pdf]

5-year mortality<br>sPAP ≥ 50 mmHg<br>Cox Regression Analysis	Univariate		Multivariate	
	**Hazard Ratio (95% CI)**	**p-value**	**Hazard Ratio (95% CI)**	**p-value**
Age	0.951 (0.713 - 1.268)	0.732		
Gender (male)	2.455 (1.240 - 4.861)	0.010	7.609 (2.857 - 20.268)	< 0.001
Height	1.142 (0.814 - 1.601)	0.443		
Weight	1.076 (0.721 - 1.604)	0.721		
BMI	0.989 (0.632 - 1.548)	0.961		
NYHA ≥ III	0.814 (0.324 - 2.041)	0.661		
STS-Score	0.904 (0.518 - 1.579)	0.723		
Diabetes mellitus	0.860 (0.404 - 1.829)	0.696		
Arterial Hypertension	1.160 (0.508 - 2.650)	0.724		
CVD	0.866 (0.443 - 1.693)	0.674		
Previous myocardial infarction	5.623 (1.680 - 18.815)	0.005	16.235 (3.596 - 73.300)	< 0.001
Atrial fibrillation	0.441 (0.217 - 0.896)	0.024	0.384 (0.156 - 0.942)	0.037
Previous cardiac surgery	4.579 (1.864 - 11.247)	0.001	2.128 (0.570 - 7.950)	0.262
Pacemaker (before TAVR)	0.571 (0.078 - 4.167)	0.580		
Malignancy	0.440 (0.135 - 1.434)	0.173		
Stroke (before TAVR)	1.061 (0.325 - 3.461)	0.921		
PAOD	0.046 (0.000 - 27.882)	0.346		
COPD	0.850 (0.300 - 2.404)	0.759		
LVEF	0.890 (0.684 - 1.159)	0.387		
LVEDD	1.053 (0.679 - 1.632)	0.818		
IVSd	0-917 (0.662 - 1.272)	0.605		
AV Vmax	0.452 (0.241 - 0.846)	0.013	0.962 (0.293 - 3.162)	0.949
AV dpmax	0.736 (0.552 - 0.981)	0.037	0.524 (0.377 - 0.729)	< 0.001
AV dpmean	0.834 (0.601 - 1.156)	0.276		
TAPSE	1.325 (0.844 - 2.080)	0.222		
AVI ≥ II°	0.910 (0.351 - 2.358)	0.847		
MVI ≥ II°	0.997 (0.516 - 1.924)	0.992		
TVI ≥ II°	0.585 (0.286 - 1.195)	0.141		
Creatinine	1.000 (0.862 - 1.160)	0.996		
BNP	0.978 (0.737 - 1.296)	0.875		
Hkt	0.834 (0.604 - 1.151)	0.270		
Hb	0.826 (0.598 -1.139)	0.244		
CK	1.223 (0.343 - 4.360)	0.756		
Pacemaker (after TAVR)	2.071 (0.996 - 4.307)	0.051	4.413 (1.766 - 11.030)	0.001
Vascular complications	0.547 (0.131 - 2.276)	0.407		
Stroke (after TAVR)	2.709 (0.643 - 11.418)	0.174		
