# Supplementary material for: Elevated systolic pulmonary artery pressure is a substantial predictor of increased mortality after transcatheter aortic valve replacement in males, not in females
Source: Clin Res Cardiol. 2023 Sep 26;113(1):138–55. doi: 10.1007/s00392-023-02307-z (PMC10808322; doi:10.1007/s00392-023-02307-z)
Supplement: Supplementary file 2 — Supplementary file2 (PDF 115 KB) [file 392_2023_2307_MOESM2_ESM.pdf]

5-year mortality<br>sPAP ≥ 40 mmHg<br>Cox Regression Analysis	Univariate		Multivariate	
	**Hazard Ratio (95% CI)**	**p-value**	**Hazard Ratio (95% CI)**	**p-value**
Age	1.030 (0.809 - 1.311)	0.811		
Gender (male)	2.350 (1.401 - 3.942)	0.001	2.555 (1.416 - 4.613)	0.002
Height	1.257 (0.970 - 1.629)	0.084	0.726 (0.486 - 1.085)	0.119
Weight	1.136 (0.874 - 1.477)	0.341		
BMI	1.041 (0.785 - 1.380)	0.781		
NYHA ≥ III	1.375 (0.697 - 2.715)	0.359		
STS-Score	0.930 (0.598 - 1.446)	0.748		
Diabetes mellitus	0.786 (0.441 - 1.402)	0.415		
Arterial Hypertension	1.031 (0.538 - 1.973)	0.927		
CVD	0.829 (0.497 - 1.381)	0.471		
Previous myocardial infarction	1.011 (0.367 - 2.781)	0.983		
Atrial fibrillation	0.703 (0.422 - 1.171)	0.176		
Previous cardiac surgery	5.482 (2.452 - 12.257)	< 0.001	8.431 (3.200 - 22.212)	< 0.001
Pacemaker (before TAVR)	1.064 (0.387 - 2.928)	0.905		
Malignancy	0.978 (0.511 - 1.872)	0.946		
Stroke (before TAVR)	1.405 (0.606 - 3.257)	0.428		
PAOD	0.734 (0.267 - 2.020)	0.550		
COPD	1.346 (0.685 - 2.644)	0.388		
LVEF	0.800 (0.651 - 0.982)	0.033	0.841 (0.657 - 1.077)	0.170
LVEDD	1.053 (0.777 - 1.427)	0.741		
IVSd	0.821 (0.637 - 1.059)	0.129		
AV Vmax	0.828 (0.630 - 1.088)	0.175		
AV dpmax	0.785 (0.605 - 1.020)	0.070	0.755 (0.537 - 1.061)	0.106
AV dpmean	0.897 (0.698 - 1.153)	0.397		
TAPSE	1.105 (0.786 - 1.553)	0.567		
AVI ≥ II°	0.973 (0.491 - 1.932)	0.939		
MVI ≥ II°	1.165 (0.699 - 1.942)	0.557		
TVI ≥ II°	0.800 (0.458 - 1.399)	0.434		
Creatinine	1.014 (0.875 - 1.174)	0.856		
BNP	1.102 (0.905 - 1.341)	0.333		
Hkt	0.886 (0.697 - 1.125)	0.320		
Hb	0.910 (0.719 - 1.151)	0.429		
CK	1.186 (0.858 - 1.639)	0.302		
Pacemaker (after TAVR)	1.441 (0.835 - 2.485	0.189		
Vascular complications	1.009 (0.460 - 2.213)	0.982		
Stroke (after TAVR)	3.579 (0.865 - 14.811)	0.078	2.397 (0.402 - 14.306)	0.338
