# Supplementary material for: Elevated systolic pulmonary artery pressure is a substantial predictor of increased mortality after transcatheter aortic valve replacement in males, not in females
Source: Clin Res Cardiol. 2023 Sep 26;113(1):138–55. doi: 10.1007/s00392-023-02307-z (PMC10808322; doi:10.1007/s00392-023-02307-z)
Supplement: Supplementary file 3 — Supplementary file3 (PDF 116 KB) [file 392_2023_2307_MOESM3_ESM.pdf]

**3-year mortality sPAP ≥ 50 mmHg Cox Regression Analysis**	**Univariate**		**Multivariate**	
	**Hazard Ratio (95% CI)**	**p-value**	**Hazard Ratio (95% CI)**	**p-value**
Age	0.974 (0.696 - 1.363)	0.878		
Gender (male)	4.303 (1.724 - 10.740)	0.002	17.035 (3.857 - 75.246)	< 0.001
Height	1.432 (0.958 - 2.139)	0.080	0.687 (0.377 - 1.251)	0.219
Weight	1.292 (0.818 - 2.040)	0.272		
BMI	1.007 (0.594 - 1.707)	0.979		
NYHA ≥ III	0.809 (0.271 - 2.417)	0.705		
STS-Score	0.848 (0.438-1.645)	0.627		
Diabetes mellitus	0.777 (0.312 - 1.936)	0.588		
Arterial Hypertension	2.102 (0.631 - 7.004)	0.226		
CVD	0.891 (0.404 - 1.964)	0.775		
Previous myocardial infarction	3.722 (0.874 - 15.860)	0.076	21.218 (3.294 - 136.692)	0.001
Atrial fibrillation	0.464 (0.202 - 1.069)	0.071	0.727 (0.395 - 1.338)	0.306
Previous cardiac surgery	5.768 (2.263 - 14.702)	< 0.001	2.958 (0.801 - 10.917)	0.104
Pacemaker (before TAVR)	0.782 (0.106 - 5.777)	0.810		
Malignancy	0.416 (0.098 - 1.759)	0.233		
Stroke (before TAVR)	0.979 (0.231 - 4.144)	0.977		
PAOD	0.046 (0.000 - 128.456)	0.447		
COPD	1.268 (0.437 - 3.682)	0.663		
LVEF	0.898 (0.655 - 1.230)	0.501		
LVEDD	1.047 (0.616 - 1.778)	0.866		
IVSd	1.054 (0.710 - 1.564)	0.794		
AV Vmax	0.401 (0.201 - 0.799)	0.009	0.313 (0.144 - 0.678)	0.003
AV dpmax	0.773 (0.555 - 1.075)	0.126		
AV dpmean	0.763 (0.523 - 1.113)	0.161		
TAPSE	1.330 (0.761 - 2.325)	0.316		
AVI ≥ II°	0.738 (0.220 - 2.475)	0.623		
MVI ≥ II°	0.895 (0.411 - 1.949)	0.780		
TVI ≥ II°	0.401 (0.161 - 1.000)	0.050	0.800 (0.269 - 2.379)	0.688
Creatinine	0.766 (0.323 - 1.820)	0.546		
BNP	1.024 (0.746 - 1.405)	0.885		
Hkt	0.932 (0.642 - 1.351)	0.709		
Hb	0.892 (0.610 - 1.303)	0.554		
CK	0.641 (0.130 - 3.165)	0.585		
Pacemaker (after TAVR)	2.293 (0.993 - 5.294)	0.052	5.517 (1.873 - 16.254)	0.002
Vascular complications	0.374 (0.051 - 2.763)	0.335		
Stroke (after TAVR)	1.424 (0.193 - 10.525)	0.729		
