# Supplementary material for: Elevated systolic pulmonary artery pressure is a substantial predictor of increased mortality after transcatheter aortic valve replacement in males, not in females
Source: Clin Res Cardiol. 2023 Sep 26;113(1):138–55. doi: 10.1007/s00392-023-02307-z (PMC10808322; doi:10.1007/s00392-023-02307-z)
Supplement: Supplementary file 4 — Supplementary file4 (PDF 116 KB) [file 392_2023_2307_MOESM4_ESM.pdf]

3-year mortality<br>sPAP ≥ 40 mmHg<br>Cox Regression Analysis	Univariate		Multivariate	
	**Hazard Ratio (95% CI)**	**p-value**	**Hazard Ratio (95% CI)**	**p-value**
Age	0.952 (0.712 - 1.275)	0.743		
Gender (male)	4.007 (1.911 - 8.401)	< 0.001	4.210 (1.843 - 9.618)	0.001
Height	1.525 (1.095 - 2.124)	0.012	0.771 (0.470 - 1.264)	0.302
Weight	1.333 (0.972 - 1.829)	0.074	1.001 (0.660 - 1.517)	0.998
BMI	1.107 (0.781 - 1.568)	0.568		
NYHA ≥ III	1.433 (0.607 - 3.380)	0.412		
STS-Score	0.965 (0.575 - 1.620)	0.894		
Diabetes mellitus	0.742 (0.354 - 1.554)	0.428		
Arterial Hypertension	1.520 (0.596 - 3.874)	0.380		
CVD	0.841 (0.446 - 1.588)	0.594		
Previous myocardial infarction	0.796 (0.192 - 3.296)	0.753		
Atrial fibrillation	0.734 (0.389 - 1.386)	0.340		
Previous cardiac surgery	7.137 (3.095 - 16.455)	< 0.001	5.231 (2.143 - 12.769)	< 0.001
Pacemaker (before TAVR)	0.815 (0.197 - 3.377)	0.778		
Malignancy	1.139 (0.526 - 2.467)	0.741		
Stroke (before TAVR)	1.459 (0.520 - 4.095)	0.473		
PAOD	0.896 (0.276 - 2.902)	0.854		
COPD	1.498 (0.664 - 3.380)	0.331		
LVEF	0.743 (0.580 - 0.951)	0.019	0.976 (0.720 - 1.324)	0.877
LVEDD	1.045 (0.714 - 1.529)	0.821		
IVSd	0.834 (0.606 - 1.148)	0.267		
AV Vmax	0.767 (0.543 - 1.083)	0.132		
AV dpmax	0.782 (0.575 - 1.065)	0.119		
AV dpmean	0.759 (0.550 - 1.048)	0.094	0.828 (0.600 - 1.143)	0.251
TAPSE	1.114 (0.727 - 1.706)	0.620		
AVI ≥ II°	0.915 (0.379 - 2.210)	0.844		
MVI ≥ II°	1.042 (0.546 - 1.986)	0.901		
TVI ≥ II°	0.619 (0.295 - 1.300)	0.205		
Creatinine	0.810 (0.401 - 1.637)	0.557		
BNP	1.137 (0.913 - 1.415)	0.252		
Hkt	0.938 (0.696 - 1.263)	0.673		
Hb	0.918 (0.684 - 1.232)	0.570		
CK	0.918 (0.465 - 1.810)	0.804		
Pacemaker (after TAVR)	1.513 (0.772 - 2.966)	0.228		
Vascular complications	0.620 (0.191 - 2.008)	0.425		
Stroke (after TAVR)	1.844 (0.253 - 13.429)	0.546		
