# Supplementary material for: Elevated systolic pulmonary artery pressure is a substantial predictor of increased mortality after transcatheter aortic valve replacement in males, not in females
Source: Clin Res Cardiol. 2023 Sep 26;113(1):138–55. doi: 10.1007/s00392-023-02307-z (PMC10808322; doi:10.1007/s00392-023-02307-z)
Supplement: Supplementary file 5 — Supplementary file5 (PDF 116 KB) [file 392_2023_2307_MOESM5_ESM.pdf]

1-year mortality<br>sPAP ≥ 50 mmHg<br>Cox Regression Analysis	Univariate		Multivariable	
	**Hazard Ratio (95% CI)**	**p-value**	**Hazard Ratio (95% CI)**	**p-value**
Age	0.796 (0.550 - 1.152)	0.226		
Gender (male)	5.885 (1.701 - 20.356)	0.005	77.954 (5.268 - 1153.475)	0.002
Height	1.697 (1.043 - 2.763)	0.033	0.973 (0.234 - 4.048)	0.970
Weight	1.704 (1.014 - 2.862)	0.044	1.183 (0.536 - 2.611)	0.677
BMI	1.285 (0.718 - 2.299)	0.398		
NYHA ≥ III	0.939 (0.235 - 3.756)	0.929		
STS-Score	0.665 (0.299 - 1.476)	0.316		
Diabetes mellitus	0.755 (0.248 - 2.293)	0.620		
Arterial Hypertension	2.100 (0.483 - 9.133)	0.323		
CVD	0.877 (0.340 - 2.263)	0.786		
Previous myocardial infarction	5.259 (1.195 - 23.142)	0.028	34.232 (4.152 - 282.267)	0.001
Atrial fibrillation	0.311 (0.102 - 0.946)	0.040	0.282 (0.067 - 1.191)	0.085
Previous cardiac surgery	5.567 (1.970 - 15.730)	0.001	0.721 (0.127 - 4.089)	0.712
Pacemaker (before TAVR)	0.046 (0.000 - 895.290)	0.542		
Malignancy	0.301 (0.040 - 2.265)	0.244		
Stroke (before TAVR)	0.689 (0.158 - 3.000)	0.620		
PAOD	0.046 (0.000 - 895.290)	0.542		
COPD	1.335 (0.386 - 4.615)	0.648		
LVEF	0.862 (0.593 - 1.255)	0.439		
LVEDD	0.036 (0.000 - 999540.990)	0.704		
IVSd	1.164 (0.715 - 1.893)	0.542		
AV Vmax	0.331 (0.148 - 0.737)	0.007	1.557 (0.011 - 230.508)	0.862
AV dpmax	0.700 (0.480 - 1.022)	0.065	0.394 (0.215 - 0.721)	0.003
AV dpmean	0.645 (0.407 - 1.020)	0.061	0.850 (0.292 - 2.476)	0.766
TAPSE	1.664 (0.797 - 3.474)	0.175		
AVI ≥ II°	0.756 (0.172 - 3.326)	0.711		
MVI ≥ II°	0.609 (0.229 - 1.624)	0.322		
TVI ≥ II°	0.282 (0.082 - 0.973)	0.045	1.323 (0.196 - 8.928)	0.774
Creatinine	0.744 (0.208 - 2.657)	0.649		
BNP	0.943 (0.627 - 1.419)	0.779		
Hkt	0.934 (0.604 - 1.443)	0.758		
Hb	0.975 (0.620 - 1.531)	0.911		
CK	0.636 (0.91 - 4.445)	0.648		
Pacemaker (after TAVR)	2.986 (1.154 - 7.728)	0.024	8.723 (2.135 - 35.644)	0.003
Vascular complications	0.043 (0.000 - 51.328)	0.384		
Stroke (after TAVR)	0.048 (0.000 - 7948.404)	0.620		
