# Supplementary material for: Elevated systolic pulmonary artery pressure is a substantial predictor of increased mortality after transcatheter aortic valve replacement in males, not in females
Source: Clin Res Cardiol. 2023 Sep 26;113(1):138–55. doi: 10.1007/s00392-023-02307-z (PMC10808322; doi:10.1007/s00392-023-02307-z)
Supplement: Supplementary file 6 — Supplementary file6 (PDF 116 KB) [file 392_2023_2307_MOESM6_ESM.pdf]

1-year mortality<br>sPAP ≥ 40 mmHg<br>Cox Regression Analysis	Univariate		Multivariate	
	Hazard Ratio (95% CI)	p-value	Hazard Ratio (95% CI)	p-value
Age	0.813 (0.575 - 1.148)	0.239		
Gender (male)	5.477 (1.879 - 15.965)	0.002	9.546 (2.218 - 41.096)	0.002
Height	1.676 (1.091 - 2.577)	0.019	1.026 (0.458 - 2.295)	0.951
Weight	1.589 (1.078 - 2.342)	0.019	1.082 (0.659 - 1.776)	0.755
BMI	1.321 (0.870 - 2.004)	0.191		
NYHA ≥ III	1.371 (0.459 - 4.092)	0.572		
STS-Score	1.098 (0.612 - 1.969)	0.754		
Diabetes mellitus	0.852 (0.340 - 2.133)	0.732		
Arterial Hypertension	1.514 (0.453 - 5.059)	0.500		
CVD	0.871 (0.384 - 1.971)	0.741		
Previous myocardial infarction	1.405 (0.331 - 5.959)	0.645		
Atrial fibrillation	0.607 (0.262 - 1.407)	0.245		
Previous cardiac surgery	5.823 (2.179 - 15.560)	< 0.001	3.863 (1.291 - 11.555)	0.016
Pacemaker (before TAVR)	0.691 (0.094 - 5.111)	0.718		
Malignancy	0.882 (0.303 - 2.569)	0.818		
Stroke (before TAVR)	2.464 (0.845 - 7.183)	0.099	2.048 (0.575 - 7.296)	0.269
PAOD	0.479 (0.065 - 3.544)	0.471		
COPD	1.741 (0.653 - 4.641)	0.267		
LVEF	0.695 (0.514 - 0.940)	0.018	0.858 (0.548 - 1.344)	0.504
LVEDD	0.755 (0.365 - 1.565)	0.450		
IVSd	0.960 (0.630 - 1.463)	0.851		
AV Vmax	0.723 (0.466 - 1.122)	0.148		
AV dpmax	0.704 (0.479 - 1.034)	0.074	0.885 (0.331 - 2.364)	0.808
AV dpmean	0.672 (0.436 - 1.036)	0.072	0.707 (0.439 - 1.137)	0.152
TAPSE	1.022 (0.563 - 1.856)	0.943		
AVI ≥ II°	0.474 (0.110 - 2.042)	0.316		
MVI ≥ II°	0.767 (0.320 - 1.836)	0.551		
TVI ≥ II°	0.546 (0.205 - 1.454)	0.226		
Creatinine	0.821 (0.346 - 1.944)	0.653		
BNP	1.060 (0.776 - 1.447)	0.715		
Hkt	1.023 (0.703 - 1.489)	0.904		
Hb	0.990 (0.679 - 1.442)	0.958		
CK	0.537 (0.107 - 2.710)	0.452		
Pacemaker (after TAVR)	1.996 (0.882 - 4.518)	0.097	1.942 (0.798 - 4.727)	0.144
Vascular complications	0.042 (0.000 - 10.652)	0.261		
Stroke (after TAVR)	0.049 (0.000 - 72768.288)	0.677		
